# Epigenetic Silencing of SOX15 Is Controlled by miRNAs rather than Methylation in Papillary Thyroid Cancer

**DOI:** 10.1155/2021/1588220

**Published:** 2021-09-24

**Authors:** Ahmet Ozaydin, Aysegul Soysal, Betul Seyhan, Soykan Arikan, Nejat Dalay, Nur Buyru

**Affiliations:** ^1^Department of Medical Biology, Istanbul University-Cerrahpasa, Cerrahpasa Medical Faculty, Istanbul, Turkey; ^2^Department of General Surgery, Istanbul Education and Research Hospital, Istanbul, Turkey

## Abstract

**Methods:**

In this study, qRT-PCR was used to investigate the expression levels of the *SOX15* gene and of miR-182, miR-183, miR-375, and miR-96 in thyroid tumors and adjacent noncancerous tissues. We also investigated the methylation status of the *SOX15* promoter by methylation-specific PCR in tumors and adjacent noncancerous tissues.

**Results:**

We observed a statistically significant downregulation of *SOX15* expression in tumors compared to noncancerous tissue samples. The methylation levels of tumors and matched noncancerous tissues were similar, but miR-182, miR-183, and miR-375 expression levels were elevated in tumor tissues compared to noncancerous tissue samples.

**Conclusions:**

Our results indicate that *SOX15* gene expression is associated with the pathogenesis of papillary thyroid carcinoma (PTC), and the epigenetic control of the *SOX15* gene is regulated by miRNAs rather than by promoter methylation.

## 1. Introduction

Thyroid cancer (TC) is a rare type of cancer and occurs as a consequence of environmental and genetic factors [1]. Cell types from which the tumors arise determine the type of thyroid cancer [2]. Among the four main types, papillary thyroid carcinoma (PTC) is the most common [3]. However, the molecular mechanism underlying the pathogenesis of thyroid carcinoma is still unclear.

Cancer can be described as a disease of altered gene expression. Activation or silencing of a gene may alter the overall activity of the cell. This can arise as a result of gene mutations or changes in the gene expression levels. Gene expression is primarily controlled by the specific binding of transcription factors to their target DNA sequences. The expression of genes is also controlled by epigenetic mechanisms such as DNA methylation and aberrant miRNA expression.

The SOX family genes code for transcription factors; however, they need additional partner proteins for the transcriptional regulation of their target genes [4]. All SOX proteins have the high mobility group (HMG) box DNA binding domain and are divided into 8 subgroups (A-G) according to the similarities in this domain [5, 6]. Members of the SOX family are expressed in a tissue-specific manner and play a critical role in the developmental processes [7]. It is well known that developmental proteins also play crucial roles in the tumor formation.

On the other hand, SOX proteins, unlike other transcription factors, bind to the minor groove of DNA and this interaction brings regulatory elements close to each other [8, 9]. Therefore, in addition to transcriptional regulation, they also act as modulators of the chromatin structure [10]. The sequences outside the HMG box also facilitate interactions between the SOX proteins and influence their specific binding properties [11].

*SOX15* is the only member of the SOXG group, and its HMG domain has a unique structure [12]. The human *SOX15* gene is universally expressed in different tissues and has been mapped to the 17p13 region [13, 14]. Among the SOX family members, *SOX15* function in cell biology and development is the least understood. Thu et al. [15] reported for the first time that *SOX15* is downregulated in pancreatic ductal adenocarcinoma (PDAC) as a result of promoter hypermethylation. Subsequently, its downregulation has been associated with the development and progression of different types of human malignancies [15–19]. As mentioned above, there is only one study in the literature investigating the *SOX15* gene in association with promoter methylation and copy number alterations [15]. However, epigenetic regulation of gene expression via miRNA molecules is an equally important mechanism. MicroRNAs (miRNAs) are endogenous noncoding RNA molecules, about 18-26 nucleotides in length, which bind to target mRNAs and posttranscriptionally regulate their expression [20]. Recent reports have indicated that deregulation of miRNAs is associated with the development and progression of various cancers including thyroid carcinoma [21, 22]. A significant number of studies have shown that miRNAs have important functions in thyroid cancer. Zhu et al. [23] reported that miR-182 exerts an oncogenic effect in thyroid cancer by downregulating CHL1 expression. More recently, it has also been reported that increased miRNA expression may have clinical and prognostic significance in thyroid cancer [21]. As a result of TargetScan database analysis, we identified a miR-96 target sequence on the *SOX15* mRNA. miR-182 and miR-96 are found in the same miRNA gene cluster together with miR-183 [24]. A recent pseudogene-gene (PGG) functional association analysis indicated that miR-375 may also regulate *SOX15* expression in different cancer types [25].

However, the role of *SOX15* and its association with miRNAs in thyroid cancer has not been investigated thoroughly. There is only a single report in the literature which investigates miR-147b in thyroid carcinoma in association with *SOX15* [26].

In the present study, to understand the epigenetic regulation of *SOX15*, we focused to investigate *SOX15* expression in association with promoter methylation and expression of four different miRNAs in thyroid carcinoma.

## 2. Materials and Methods

### 2.1. Patients

Primary thyroid tumors and adjacent nonmalignant tissue samples were collected from 52 patients, prior to any treatment at the Istanbul Education and Research Hospital between April 2016 and May 2017. The study was approved by the Ethics Committee of Istanbul University-Cerrahpasa, Cerrahpasa Medical Faculty (No: 71305). Signed informed consent was obtained from all patients before sample collection.

Table 1 lists the clinicopathological features of the patients. Pathological analysis was performed at the Pathology Department of the Istanbul Education and Research Hospital.

### 2.2. Methylation-Specific Polymerase Chain Reaction

Genomic DNA was obtained using the High Pure PCR Template Preparation Kit (Roche, Germany). After spectrophotometric quantitation, 500 ng of genomic DNA was bisulphide-treated using the EZ DNA Methylation-Gold Kit (Zymo Research, CA, USA) and finally resuspended in 10 *μ*l TE buffer. PCR was performed in 25 *μ*l volume containing 200 ng of modified DNA as template, 10x buffer, 100 mM dNTP, 10 pmol of each primer, and 5 U/*μ*l AmpliTaq Gold DNA polymerase (ThermoFisher Scientific, MA, USA). Primers of methylated and unmethylated sequences were designed by the MethPrimer methylation analysis software and are listed in Table 2. The PCR products were directly loaded onto 2% agarose gels and analyzed using the Bio1D software (Vilber Lourmat, France) under UV light. The volume/area ratios were calculated to determine the methylation level of the *SOX15* gene.

### 2.3. Reverse Transcription and Quantitative RT-PCR

Total RNA was isolated from tissue samples by using the PureLink™ RNA Mini Kit (ThermoFisher Scientific, MA, USA) according to the manufacturer's instructions. Reverse transcription was performed using 300 ng of total RNA and the Reverse Aid First-Strand cDNA synthesis kit (ThermoFisher Scientific, MA, USA). Expression levels of the *SOX15* gene were analyzed by qRT-PCR using the SYBR green and LightCycler 480 system (Roche Diagnostics, Germany).

*β*2M was used as the reference to normalize the mRNA levels of each sample for quantification. CT values of the target and reference genes in tumor and normal thyroid tissues were analyzed by the LightCycler Software. Expression changes were determined by the relative mRNA levels using the 2^-∆∆Ct^ method [27].

### 2.4. miRNA Quantification

TaqMan microRNA RT kit (ThermoFisher Scientific, MA, USA) was used for cDNA synthesis according to the manufacturer's instructions. The expression levels of miRNAs were analyzed with TaqMan MicroRNA Assay (hsa-mir-182-5p ID: 002334, hsa-mir-183-5p ID: 002269, hsa-mir-375-3p ID: 000564, and hsa-mir96-3p ID: 002140) (ThermoFisher Scientific, MA, USA). qRT-PCR amplification was performed using the protocol for TaqMan™ Small RNA Assays user guide (Publication Number 4364031, Revision Date 10 December 2019 Rev. H). qRT-PCR was performed using the LightCycler 480 system (Roche Diagnostics, Mannheim, Germany). Ct values of target miRNAs were normalized to U6 small nuclear RNA (ID: 001973), and the fold changes in expression levels of each miRNA were calculated using the 2^-∆∆Ct^ method. The target and reference miRNAs were coamplified in the same reaction.

### 2.5. Statistical Analyses

Statistical analysis was performed using the SPSS 21.0 (IBM® SPSS® Statistics, IBM Corporation Somers, NY, USA) program. The paired sample *t*-test was used, and *p* < 0.05 was considered statistically significant for data showing normal distribution. The nonparametric counterpart of the paired sample *t*-test, Wilcoxon Signed Rank Test, was used for unequally distributed expression levels.

## 3. Results

**Figure 1:**
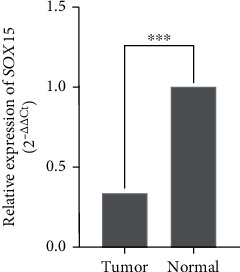
SOX15 gene expression in thyroid cancer tumor tissues compared to their normal counterparts. ^∗∗∗^*p* ≤ 0.001.

The expression level of the *SOX15* gene was analyzed in 49 pairs of thyroid tumors and adjacent noncancerous tissues. We detected the *SOX15* transcript in both tumor and normal tissue samples. However, *SOX15* gene expression was significantly downregulated in 64.6% (34/49) of TC tissues compared to their normal counterparts (Figure 1). Next, we explored the relationship between *SOX15* expression levels and the clinicopathological features of TC patients. The level of *SOX15* expression was not associated with any particular clinicopathological parameter.

One of the mechanisms which leads to downregulation of gene expression is promoter methylation. Further, we investigated the methylation status of the *SOX15* promoter (-158 to -382 region) by Methylation-Specific PCR (MSP) in 44 paired tumors and adjacent noncancerous tissues. In TC samples, 15 cases (15/44) revealed both moderately unmethylated and highly methylated forms, and 3 cases revealed both methylated and unmethylated forms. 25 of the remaining 26 samples were completely methylated, and one sample was completely unmethylated. The noncancerous adjacent counterpart of the completely unmethylated tumor sample was also completely unmethylated. 21 of the remaining noncancerous samples were completely methylated, and 22 revealed both methylated and unmethylated forms.

We did not observe statistically significant differences between the methylation levels of tumors and matched noncancerous tissues (*p* = 0.115). Our results indicate that the downregulation of the *SOX15* gene is not associated with promoter methylation (Table 3).

Thus, we concluded that a different mechanism such as miRNAs might modulate *SOX15* expression. For this purpose, we investigated the expression rates of miRNA-182, miRNA-183, miRNA-96, and miRNA-375 in 45 matched tumor and noncancerous tissue pairs. Significant differences in miRNA-182, miRNA-183, and miRNA-375 levels were observed between TC tumors and matched normal tissues. However, miRNA-96 levels were similar both in tumor and in normal tissues. For the tumor and normal tissues, the levels of miRNAs are presented in Table 4.

## 4. Discussion

Thyroid cancer is one of the most common endocrine malignancies. Although some genetic alterations such as *BRAF*, *RAS*, *CTNNB1*, *TP53*, and *EGFR* mutations have been associated with thyroid cancer, additional molecular mechanisms are thought to be involved in the formation and progression of TC [28–32]. Aberrant activation of signaling pathways is a common mechanism in human cancers. One of the important pathways is the Wnt/*β*-catenin signaling pathway which regulates cellular events such as proliferation, differentiation, and cell motility. Several secreted protein families activate or inhibit Wnt/*β*-catenin signaling. Upon activation, the Wnt/*β*-catenin pathway triggers the formation and progression of different types of human cancers [33]. Previous reports have shown that some of the SOX gene family members are negative regulators (or antagonists) of the Wnt/*β*-catenin signaling pathway [34, 35]. Thu et al. have identified *SOX15* as a negative regulator of the Wnt/*β*-catenin pathway in PDAC [16]. In contrast to other members of the SOX family, function of the *SOX15* gene is not well defined in cancer. It is well documented that *SOX15* has critical functions in myogenic differentiation [36]. Following this initial report, other studies have associated aberrant *SOX15* expression with other kinds of tumors such as gastric, endometrial, and colon cancer [15–19].

However, *SOX15* has not been investigated in detail in TC. To our knowledge, only a single study is available in the literature, which investigates *SOX15* in TC [26]. According to this report, *SOX15* is underexpressed in TC tumor cells and cell lines under the influence of miR-147b and silencing of *SOX15* via miR-147b activates the Wnt/*β*-catenin pathway.

In accordance with the previous results, we observed a significant downregulation of the *SOX15* gene in the PTC tumor samples compared to normal tissue. Our data analysis revealed that mutation is not a frequent event in *SOX15* inactivation. As a result of the multiomics approach, it has been reported that *SOX15* is inactivated by concurrent hypermethylation and DNA copy number loss in PDAC [15]. However, it should be noted that regulation of expression is also controlled by other genetic and epigenetic mechanisms. Therefore, we investigated promoter methylation of the *SOX15* gene in PTC tumor cells in association with its expression levels. In contrast to PDAC, our results indicate that downregulation of *SOX15* is not caused by promoter hypermethylation. On the other hand, increasing evidence indicates that miRNAs play important roles in gene inactivation. Accumulating data show that various miRNAs are dysregulated in thyroid carcinoma and most of these miRNAs are involved in the regulation of malignancy and metastasis in TC [21–23, 26, 37, 38]. Hitu et al. [22] have reported that 106 of 139 miRNAs which have been investigated were upregulated in PTC while 33 were downregulated. In a previous report, Zhu et al. [23] revealed that overexpression of miR-182 regulates PTC proliferation and invasion through downregulating *CHL1* expression. More recently, another report also associated miR-182 overexpression with extrathyroidal invasion, cervical lymph node metastasis, and TNM staging in PTC [38]. In accordance with these reports, we observed 3.11 times higher miR-182 levels in PTC tumor samples compared to normal tissues. However, overexpression of miR-182 was not associated with any clinicopathological characteristics of the patients. miRNAs frequently reside in clusters, and members of clusters are generally transcribed in the same direction [24]. miR-182/183/96 also are usually found as a miRNA cluster. Therefore, we investigated expression levels of miR183 and miR-96 together with miR-182 in our study cohort. Although the expression level of miR-183 increased similar to miR-182, miR-96 expression was at the same level in the tumors and normal noncancerous tissues. Our TargetScan analysis revealed that a possible target sequence for miR-96 was present in the *SOX15* gene; however, we did not observe altered miRNA-96 expression or any association between the *SOX15* and miRNA-96 expression levels. This result is in contrast to a report which suggested that high miRNA-96 expression in PTC tissue and cell lines promotes cell proliferation, migration, and invasion via downregulating the Deup1 protein expression [37]. This difference may be due to processing of tissues under different conditions. We used tissue samples as soon as they were surgically removed. It should also be noted that not all target sequences predicted by TargetScan are actually valid. Indeed, the estimates for false-positive rates for target prediction are at the level of 50% and the results of the target prediction programs are inconsistent [39–41].

miR-375 is one of the highly conserved miRNAs in humans through the evolution [42]. According to PGG network analysis, Johnson et al. have reported that differential expression of *SOX15* was associated with miR-375 expression in prostate cancer [25]. Although in our study downregulation in *SOX15* expression was not correlated with miR-375 overexpression, we detected a statistically significant increase in miR-375 expression in tumor samples compared to noncancerous ones. Our expression analysis also showed that miRNA-375 expression levels are as high as miRNA-182 and miRNA-183.

As indicated above, SOX15 has been shown to modulate the Wnt/*β*-catenin pathway [16–18]. Likewise, miR-183 has been shown to act as an important target in the regulation of the Wnt/*β*-catenin pathway in different cancer types [43–45]. On the other hand, miR-182 has been associated with tumor progression and chemoresistance in various tumors [46–48] and with suppression of apoptosis in papillary thyroid cancer [49]. miR-375 and miR-96 were shown to affect various pathways in different tumors and have been reported to regulate the PI3K/Akt pathway in thyroid cancer [50, 51].

In view of lack of studies on the function of SOX proteins in thyroid cancer, we believe that our data suggest a role for SOX15 and increased miR-182, miR-183, and miR-375 levels in the tumor samples. The results of the present study indicate that the correlation between the miRNAs and SOX15 warrants further research to reveal their role and detailed mechanism in thyroid carcinogenesis. Although investigating expression of the SOX15 protein to confirm the mRNA expression levels would corroborate our results, unfortunately, most of the samples made available for this study were not sufficient to analyze SOX15 protein expression by western blotting in matched pairs of tissue specimens. Concordance of the SOX15 mRNA expression levels with cellular protein levels in the tissue remains to be shown/confirmed. However, for SOX15, a high degree of agreement between the mRNA and protein expression has been shown in pancreatic [16] and colorectal [18] cancers as well as in gliomas [35]. Deciphering the role and mutual interaction of SOX15 with specific miRNAs will certainly help to provide further insight implicating the cellular signals and pathways involved in thyroid carcinogenesis.

## 5. Conclusion

In conclusion, our results indicate that the *SOX15* gene is associated with PTC pathogenesis and the epigenetic control of this gene is regulated by miRNAs rather than promoter methylation. The results of the study need to be verified by the analysis of a larger number of TC samples and further correlation studies of miRNAs.

## Figures and Tables

**Table 1:** Clinicopathological characteristics of patients.

Clinicopathological characteristics		*n* (%)
Sex	Female	44 (84.62)
	Male	8 (15.38)
Age	≤55	35 (67.31)
	>55	16 (30.77)
	Unknown	1 (1.92)
TNM stage	TNM1	34 (65.38)
	TNM2	7 (13.46)
	TNM3	6 (11.54)
	TNM4	3 (5.77)
	Unknown	2 (3.85)
Lymphatic invasion	Present	17 (32.69)
	Absent	34 (65.38)
	Unknown	1 (1.92)
Vascular invasion	Present	7 (13.46)
	Absent	44 (84.61)
	Unknown	1 (1.92)
Perineural invasion	Present	6 (11.54)
	Absent	45 (86.54)
	Unknown	1 (1.92)
Capsule invasion	Present	17 (32.69)
	Absent	33 (63.46)
	Unknown	2 (3.85)
Calcification	Present	26 (50)
	Absent	21 (40.38)
	Unknown	5 (9.62)
Tumor diameter	≤2	34 (65.38)
	2-4	15 (28.85)
	>4	2 (3.85)
	Unknown	1 (1.92)
Histologic type	Papillary	47 (90.38)
	Medullar	1 (1.92)
	Follicular	1 (1.92)
	Anaplastic	1 (1.92)
	Unknown	2 (3.85)

**Table 2:** Primer sequences used in this study.

Primer	Sequence
SOX15 qRT-PCR	F: 5′-CAGCTATGGCTCTTCCCACTG-3′ R: 5′-AGGGTTGTATGGAGTGGGAGA-3′
*β*2M qRT-PCR	F: 5′-CTCGCGCTACTCTCTCTTTCTGG-3′ R: 5′-GCTTACATGTCTCGATCCCACTTAA-3′
SOX15 methylated	F: 5′-TTATTCGCGTTTGGTAGTTGTC-3′ R: 5′-AAACCTTTACTTCCAACCTATTCG-3′
SOX15 unmethylated	F: 5′-GGTTTATTTGTGTTTGGTAGTTGTT-3′ R: 5′-AAACCTTTACTTCCAACCTATTCAAC-3′

**Table 3:** Correlation between SOX15 mRNA expression and promoter methylation.

		SOX15 expression *n* (%)			
		Increase	Decrease	No change	^∗^ *p*
Methylation	Increase	4 (9.1)	9 (20.5)	3 (6.8)	0.162
	Decrease	1 (2.3)	8 (18.2)	0 (0)	
	No change	5 (11.4)	13 (29.5)	1 (2.3)	

^∗^Statistical analysis was performed by using Spearman correlation.

**Table 4:** Mean (±SD) expression values of miRNA-182, miRNA-183, miRNA-96, and miRNA-375 in tumor and noncancerous tissues.

		Target Ct Mean ± SD	Reference Ct Mean ± SD	∆Ct Mean ± SD	∆∆Ct	2^-∆∆Ct^	*p*
miRNA-182	Tumor	34.46 ± 1.85	24.16 ± 1.34	10.32 ± 2.03	-1.64	3.11	^∗^ <0.001
	Noncancerous	35.74 ± 1.55	23.9 ± 1.09	11.85 ± 1.53	0	1	
miRNA-183	Tumor	33.67 ± 2.06	24.53 ± 1.35	9.14 ± 1.98	-1.27	2.41	^∗^0.003
	Noncancerous	34.59 ± 1.59	24.22 ± 1.16	10.38 ± 1.57	0	1	
miRNA-375	Tumor	32.19 ± 3.19	24.56 ± 1.37	7.63 ± 3.55	-2.04	4.1	^∗∗^0.001
	Noncancerous	33.93 ± 2.14	24.26 ± 1.19	9.67 ± 2.25	0	1	
miRNA-96	Tumor	36.47 ± 1.5	23.27 ± 1.6	12.93 ± 2.33	0.24	0.85	^∗∗^0.628
	Noncancerous	36.31 ± 1.42	23.05 ± 1.69	12.7 ± 1.79	0	1	

^∗^Statistical analyses were performed by paired sample *t*-test. ^∗∗^Statistical analyses were performed by the Wilcoxon Signed Rank Test. SD: standard deviation.

## Data Availability

Data are available on request.
